# A Prospective Cohort Study Investigating the Behavioural Development of Bitches in a Guide Dog Training Programme Neutered Prepubertally or Post-Pubertally

**DOI:** 10.3389/fvets.2022.902775

**Published:** 2022-07-07

**Authors:** Rachel Moxon, Sarah Freeman, Richard Payne, Sandra Corr, Gary C. W. England

**Affiliations:** ^1^Canine Science, Guide Dogs National Centre, Leamington Spa, United Kingdom; ^2^School of Veterinary Medicine and Science, University of Nottingham, Nottingham, United Kingdom; ^3^School of Veterinary Medicine, College of Medical, Veterinary and Life Sciences, University of Glasgow, Glasgow, United Kingdom

**Keywords:** dog, bitch, behaviour, neuter, guide dog, puberty, assistance dog

## Abstract

There are few studies that investigate the effect of neutering bitches before or after puberty. The majority of current literature examining the impact of the timing of neutering on health and behaviour has used age rather than the onset of puberty as the key variable. The aim of this prospective cohort study was to investigate the effects of timing of neutering in relation to puberty on behaviour in female dogs reared and trained in an assistance dog programme. The study examined data for bitches neutered before or after puberty to compare scores for six behavioural factors (training and obedience, aggression, fear and anxiety, excitability, attachment and attention-seeking, and social behaviour) measured at 1 and 3 years of age. Labrador and Golden Retriever crossbreed bitches were neutered before (*n* = 155) or after (*n* = 151) puberty. Neutering before or after puberty had no impact on mean scores for the six behavioural factors at 1 or 3 years of age. When examining the change in behavioural factor scores between 1 and 3 years of age, only aggression behavioural factor scores were influenced by neutering before or after puberty. Bitches neutered after puberty were less likely to have aggression factor scores that increased between 1 and 3 years of age (OR = 0.959, 90% CI = 0.924 to 0.995, *p* = 0.06). However, the majority of bitches scored “0” for aggression at both time points (indicating no aggression behaviours were observed), and the number of bitches for which scores increased between 1 and 3 years of age was low (before puberty = 20, after puberty = 9). This is consistent with very mild aggressive behaviours being observed in a small number of animals and is, therefore, of questionable concern. The results suggest that, for Labrador and Golden Retriever crossbreed bitches, neutering before or after puberty has little to no effect on future behaviour. It is recommended that decisions about the timing of neutering are not informed solely by impacts on behaviour, but that they also consider evidence relating to the impacts on bitch health and well-being.

## Introduction

Behavioural problems can lead to dogs being relinquished to animal shelters, can result in failed adoptions from shelters and can negatively impact dog well-being (1–3). Problem behaviours, or subsequent training difficulty, are the most commonly reported reasons for dogs being unsuccessful in assistance dog training programmes (4–6). Understanding the factors that affect the likelihood of behavioural problems being observed in dogs is, therefore, important, not only for maintaining the human-animal bond and for dog welfare but also to help to minimise the dogs needing to be released and rehomed from assistance dog training programmes for behaviour-related reasons.

Neutering has been suggested to impact behaviour in female dogs by many authors (6–21). However, considerable variation between study populations and methodology, alongside contradictory findings, make associations unclear for many behaviours. The effects of neutering on behaviour are also likely to differ by sex, and between breeds (8, 19, 22–24). The extent of between-study variation for studies, comparing behaviour between neutered and entire female dogs becomes apparent when considering, perhaps, one of the most well-studied behaviours in relation to neutering; aggression. Aggressive behaviours in female dogs have been reported to be unaffected by neutering by some authors (20, 23, 25, 26), whereas others report increased aggressive behaviour in neutered dogs (17, 21, 27–30), or conversely beneficial effects of neutering on aggression are also reported (18, 31, 32).

The effects of the timing of neutering on behaviour have also been examined, with the most common methodology using age at neutering rather than any assessment of the onset of puberty. Similar to findings related to behaviour in entire and neutered dogs, the reported effects of age at neutering are not consistent across behaviours or between sexes (8, 10, 15, 33). The age groups used in these studies vary; in three separate studies, data were compared for dogs neutered at 7 weeks compared to 7 months (8), dogs neutered at <24 weeks compared to 24 weeks of age or older (33), and dogs neutered at <5 and 1/2 months (approximately 24 weeks) of age compared to 5 and 1/2 months of age or older (10). Inferring effects of neutering before or after puberty from these studies is not possible, as the actual onset of puberty was not recorded and the way in which groups were selected means that, although in the younger age groupings, most dogs were likely to be prepubertal, the older age groupings would contain a mixture of dogs that were either pre- or post-pubertal at the time of neutering.

There is a lack of evidence regarding the effects of timing of neutering in relation to puberty on female dog behaviour. The only current published study in which puberty was defined reported no differences in urination behaviour and owner-reported behaviour for 58 Labrador Retriever bitches (20). Despite the lack of evidence, many authors have implied in review papers and conference proceedings that studies using the onset of puberty have been conducted (34–41). However, these appear to be based upon apparent misinterpretation of studies that investigate the impacts of neutering at different ages. Large-scale longitudinal studies, which include valid information on age at puberty for individual dogs and that measure specific behaviours over time, have been recommended to improve understanding of the effects of neutering before or after puberty on behaviour (6, 17, 41).

The lack of available evidence makes for difficult decision-making for working or assistance dog organisations, with a policy of neutering their dogs. The aim of this study was to investigate the effects of timing of neutering in relation to puberty (rather than age) on behaviour of bitches from a guide dog programme in a prospective cohort study design. Based on published studies, which have reported impacts of neutering age on behaviour, the study hypothesis was that the neutering of bitches prepubertally or post-pubertally can have different effects on their behavioural development. The study examined data for bitches neutered before or after puberty to compare behavioural factor scores measured using questions from the Canine Behavioural Assessment and Research Questionnaire (C-BARQ).

## Materials and Methods

The study involving animal and human participants was reviewed and approved by the University of Nottingham, School of Veterinary Medicine and Science Committee for Animal Research and Ethics. The ethical review number is 501 120106. The aspect of the study involving behaviour questionnaires is considered a business as usual activity and did not require specific human ethics approval. The standing agreement between Guide Dogs and persons caring for its dogs is such that should any issues come to light, for instance, *via* a survey, dog and human welfare would be followed up. All study animals belonged to Guide Dogs or were rehomed at the time of behavioural data collection. Any persons that had opted out of contact for research were not contacted to participate.

### Study Design

A prospective cohort study was undertaken using 306 bitches born between 22 February 2012 and 9 August 2015 in a guide dog programme. The bitches were neutered either prepubertally (at 6 months of age, PPN) or post-pubertally (after their first oestrus, Control). Ovariohysterectomy by celiotomy was used to neuter all the bitches. Surgeries were performed at one of four UK veterinary practices, all four veterinary practices performed both pre- and post-pubertal neutering surgeries. Data were gathered to examine bitch behaviour reported by handlers at 1 and 3 years of age.

### Study Setting

Within the guide dog programme, dogs are placed into puppy raising in volunteer family homes between 7 and 8 weeks of age, where they remain until commencing early training at ~14 months of age; after which, they undergo a period of advanced training. During training stages, dogs are housed in kennels or with boarders in their homes. By 3 years of age, dogs have either qualified as guide dogs and are living with their guide dog owners or have been withdrawn from the training programme and are either rehomed with their new owners (rehomers), working as buddy dogs or have been transferred to an alternative working home. All dog movements through the stages of the training programme are recorded in the guide dog programme's electronic database.

Initial behaviour questionnaires were sent by email when the bitches were 1 year old, for completion within a 2-week period. At the 3-year time point, the owners of working 3-year-old guide dogs were contacted by telephone and invited to take part, and questionnaires were completed by telephone or by email, depending on preference. In the case of the bitches that were withdrawn and rehomed by 3 years of age, the questionnaires were sent by email to the bitches' new owners for completion and returned by email. The bitches that had been rehomed in alternative working homes (*n* = 8) were excluded.

### Study Participants

The study population included 306 bitches from five different crossbreeds, allocated to two groups: prepubertally neutered (PPN, *n* = 155) and Control (*n* = 151; Table 1). All second-generation backcross bitches (those with a sire of one breed and a crossbreed dam) were grouped into one breed group for analysis.

**Table 1:** The number of bitches of each breed in the final study population, investigating the effect of neutering before (PPN) or after (Control) puberty.

**Breed**	**PPN** **bitches**	**Control** **bitches**
Golden Retriever cross Labrador	100	104
Golden Retriever cross (Labrador cross Golden Retriever)	0	1
Labrador cross Golden Retriever	24	19
Labrador cross (Golden Retriever cross Labrador)	28	22
Labrador cross (Labrador cross Golden Retriever)	3	5
Total	155	151

*Sire breed is written first*.

1-year behaviour questionnaires were sent to puppy raisers (*n* = 303) or rehomers (*n* = 2) for 305 bitches (one bitch was in buddy dog training and did not have a questionnaire sent). For the 3-year behaviour questionnaires, the owners of 243 bitches were invited to participate; 151 guide dog owners (by telephone) and 91 rehomers, of which 25 had also been the bitch's puppy raiser, and one volunteer boarder (by email). Questionnaires that were completed outside of the 2-week window were excluded from analysis.

### Variables and Data Sources

A master version of the C-BARQ was obtained from the University of Pennsylvania in 2011. This 101-item questionnaire (42) was shortened to suit the study population in consultation with Guide Dogs' training and behaviour and dog health teams. Some questions from the original C-BARQ were combined, for instance, combining separate questions relating to responses to adults and children, or to male and female dogs, to one question asking about responses to a person, or to a dog, as per the mini-C-BARQ (43). The final questionnaire contained 42 questions, which were grouped into six factors: 1) training and obedience, 2) aggression, 3) fear and anxiety, 4) excitability, 5) attachment and attention-seeking, and 6) social behaviour (termed “Miscellaneous” in the original C-BARQ – see Supplementary File 1). There was an additional question included (Q43), asking whether the bitches ever raised their legs during urination.

The questionnaire was designed to be completed by the handlers to gather information about the behaviours observed during the previous few months. Each question was completed by selecting an option on a rating scale. Responses to items in the training and obedience, attachment and attention-seeking, and social behaviour factors were either “Never,” “Seldom,” “Sometimes,” “Usually” or “Always.” Responses that could be selected for the aggression, fear and anxiety, and excitability factors were numerical on a scale from “0” to “4” where “0” represented a behaviour that had not been observed, and “4” represented a high severity or frequency of the behaviour (42). High scores represented less desirable behaviour for all items, except four training and obedience items, which were reversed prior to analysis. Outcome variables were the six factor scores at 1 and 3 years of age and the change in factor scores.

### Bias and Study Size

Confounding factors were included in statistical models to account for their effects when examining the main effect of trial group on the dependent variables. The dogs in this study were of similar breeds. However, breed was included in statistical models to control for the potential effect on other variables, as C-BARQ scores have been shown to vary between breeds of dogs (22, 44–47). The region of the UK and the Guide Dogs training location were included in all models to account for any variation resulting from dogs being trained by staff in different areas of the UK. Behavioural assessments and questionnaires completed for animals are known to be subject to variability caused by different observers or participants (48, 49). All returned 1-year questionnaires were completed by puppy raisers. For analysis of 3-year behaviour questionnaires, the person completing the questionnaire (the guide dog owner, rehomer, boarder or puppy raiser) was included in the models. The Control bitches were neutered after their first season, and season dates varied amongst Control bitches. The number of days from neutering surgery was included in the models as a continuous covariate, with “0” representing the day of neutering to account for any variation in behaviour that may be caused by some bitches having been neutered closer to the date of questionnaire completion than others.

Study size was determined by the availability of a cohort of bitches produced by Guide Dogs that were placed in puppy raising during the recruitment period and taken to one of the four national veterinary practices for neutering surgery. Values of alpha were determined using sensitivity analysis in G^*^Power (http://www.gpower.hhu.de/) for each analysis to minimise the likelihood of Types I and II errors and in order to use values of alpha appropriate for the study's fixed sample size (1-year questionnaires, *n* = 274; 3-year questionnaires, *n* = 193; change in behaviour scores between 1 and 3-year questionnaires, *n* = 171) and size of effect that would be expected to be significant. The mean and confidence intervals (CI) were reported where appropriate.

### Quantitative Variables and Statistical Methods

Categorical covariates included in all statistical models were examined using descriptive statistics, and any which had less than five bitches were identified and grouped.

#### Behavioural Factor Internal Consistency

Internal consistency for the six behavioural factors was tested using Cronbach's alpha, which was reported alongside the mean inter-item correlation matrix (IICM). Data for all bitches from 1 and 3 years of age were combined. A Cronbach's alpha of >0.70 was considered to indicate good internal reliability. However, as all scales had fewer than 10 items, a Cronbach's alpha of >0.5 was considered acceptable in this case (50, 51).

#### Behavioural Factor Scores

The mean score for each behavioural factor was calculated for each bitch and used for statistical analysis (52). Mean scores from 1 year of age were subtracted from mean scores at 3 years of age to calculate the change in behavioural factor scores for each bitch between the two time points. Differences between −0.1 and 0.1 were classed as “No change.” An increase in the mean factor score represented a move towards less desirable behaviour from 1 to 3 years of age, whilst a decrease in the mean factor score represented a move to more desirable behaviour. For 3-year questionnaires, the one boarder response was grouped with rehomer responses for analysis.

Scores for items in training and obedience, attachment and attention-seeking, and social behaviour factors were determined by converting the word responses to numerical scores from “0” to “4” where “0” represented the most desirable behaviour and “4” the least desirable behaviour for each question. Scatterplots were produced to visualise any relationship between days from neutering surgery and mean factor scores and relationships were examined using Spearman's rank correlation coefficients. At 1 year of age, differences in mean factor scores for Control bitches that had and had not been neutered at the time of questionnaire completion were examined using Mann–Whitney U tests with Bonferroni correction applied.

For all behavioural factors for 1-year questionnaires, alpha and beta were 9 and 5%, respectively, for a sample size of 274 dogs, with an effect size of 0.405. For all behavioural factors for 3-year questionnaires, alpha and beta were 10 and 5%, respectively, for a sample size of 193 dogs, with an effect size of 0.476. For the change in behavioural factor scores between 1 and 3-year questionnaires, alpha and beta were 10 and 5%, respectively, for a sample size of 171 dogs, with an effect size of 0.506. Differences in mean factor scores between PPN and Control bitches for 1 and 3-year questionnaires were examined using general (for training and obedience and attachment and attention-seeking) or generalised (for a gamma distribution for fear and anxiety, excitability, and social behaviour) linear models, depending on score distribution and the model fit. Change in mean factor scores between 1 and 3 years of age was examined using general (for training and obedience, excitability, attachment and attention-seeking, and social behaviour) or generalised (for fear and anxiety and aggression) linear models, depending on score distribution and the model fit. Standardised residuals were examined for general linear models. Where significant effects of confounding factors with more than two groups were identified from general linear models, Tukey's HSD *post-hoc* tests were applied. Due to the highly skewed distribution of Aggression scores for 1 and 3-year questionnaires (median aggression factor scores were 0), data were converted to binary responses where “0” represented no aggression observed, and scores of “1” or more represented any aggression (43, 53, 54) and were analysed using a generalised linear model with a logit link function.

Bitch breed (three groups) and trial group were included as fixed factors in all models. For models examining behaviour scores at 1-year of age, the puppy raising region (Scotland and Northern Ireland, North, Wales and West, South and East) was included as an additional fixed factor, and age at questionnaire completion and the number of days from neutering surgery were included as covariates. For models examining behaviour at 3 years of age, the person completing the questionnaire (the guide dog owner, rehomer, puppy raiser-rehomer) was included as an additional fixed factor, and age at questionnaire completion and the number of days from neutering surgery were included as covariates. For the analysis of the change in behaviour scores between 1 and 3 years of age, inclusion of covariates for age and the number of days since neutering surgery was not possible. The person completing the questionnaires for dogs that were 3 years of age was included as a fixed factor. All the models were fitted using IBM SPSS Statistics for Windows, version 22 (IBM Corp., Armonk, N.Y., USA).

The direction of the change in scores for each behavioural factor between time points was presented graphically by the trial group. The number of behavioural factors (out of six) for each bitch with scores that increased, decreased or did not change was determined. This was used to categorise each bitch with an overall direction of behavioural change towards more desirable behaviours (four, five or six scores that decreased by 3 years of age, none or one that increased, and/or none to two that remained the same), less desirable behaviours (four, five or six scores that increased by 3 years of age, none or one that decreased, and/or none to two that remained the same), or mixed (between one and four scores that increased or decreased and/or between none and four scores that remained the same). The number of bitches with each overall direction of change was compared between trial groups using a chi-square test (XLStat2016, Addinsoft, USA).

#### Leg Raising When Urinating

The responses for leg raising during urination were examined independently, and data were described. No statistical analyses were performed due to low incidence of the posture being reported.

## Results

### Participants

Behaviour questionnaire data were available for 134 PPN and 140 Control bitches at 1 year of age and 102 PPN and 91 Control bitches at 3 years of age, including 171 bitches (89 PPN, 82 Control) at both time points. At 3 years of age, questionnaires were completed for 115 bitches that had qualified and were working as guide dogs, 15 of which were working with their second guide dog owner; 77 bitches that had been withdrawn, six of which were withdrawn following qualification, and one bitch that was in advanced training after being withdrawn post-qualification. In total, 195 bitches qualified as guide dogs (97 PPN, 98 Control).

At the time of 1-year behaviour questionnaire completion, all 134 PPN and 49 Control bitches were neutered, 39 had been neutered in the previous month (questionnaires completed 1 to 30 days after neutering surgery). At the time of the 3-year questionnaires, all bitches had been neutered between 617 and 972 days prior to questionnaire completion.

### Behavioural Factor Internal Consistency

Behavioural data were included for 476 completed questionnaires. Cronbach's alpha was >0.7 for three factors: fear and anxiety (0.704, IICM = 0.210), excitability (0.825, IICM = 0.486), and attachment and attention-seeking (0.708, IICM = 0.283) and was >0.5 for two factors: training and obedience (0.681, IICM = 0.269) and social behaviour (0.585, IICM = 0.158). Reliability analysis for aggression items was not possible due to the large number of bitches that had scores of “0” reported for all items (401/476).

### Behavioural Factor Scores

#### Questionnaires at 1 Year of Age

The PPN bitches were neutered between 143 and 228 days before questionnaire completion. The Control bitches were neutered 145 days before to 92 days after questionnaire completion. Spearman's rank correlation coefficients (*r*_*s*_ < 0.06 for all factors) and Scatterplots did not suggest any strong relationships between days from neutering surgery and mean behavioural factor scores; PPN and Control bitch scores were clustered in two distinct groups by days from neutering surgery (see Supplementary File 2). Following sequential Bonferroni correction, there were no differences in behaviour factor scores for any of the six behavioural factors between Control bitches that were (*n* = 49) and were not (*n* = 91) neutered at the time of behavioural questionnaire completion. Data for all Control bitches were combined and used for analysis.

##### Factors Influencing Behavioural Factor Scores at 1-Year Questionnaires

**Figure 1:**
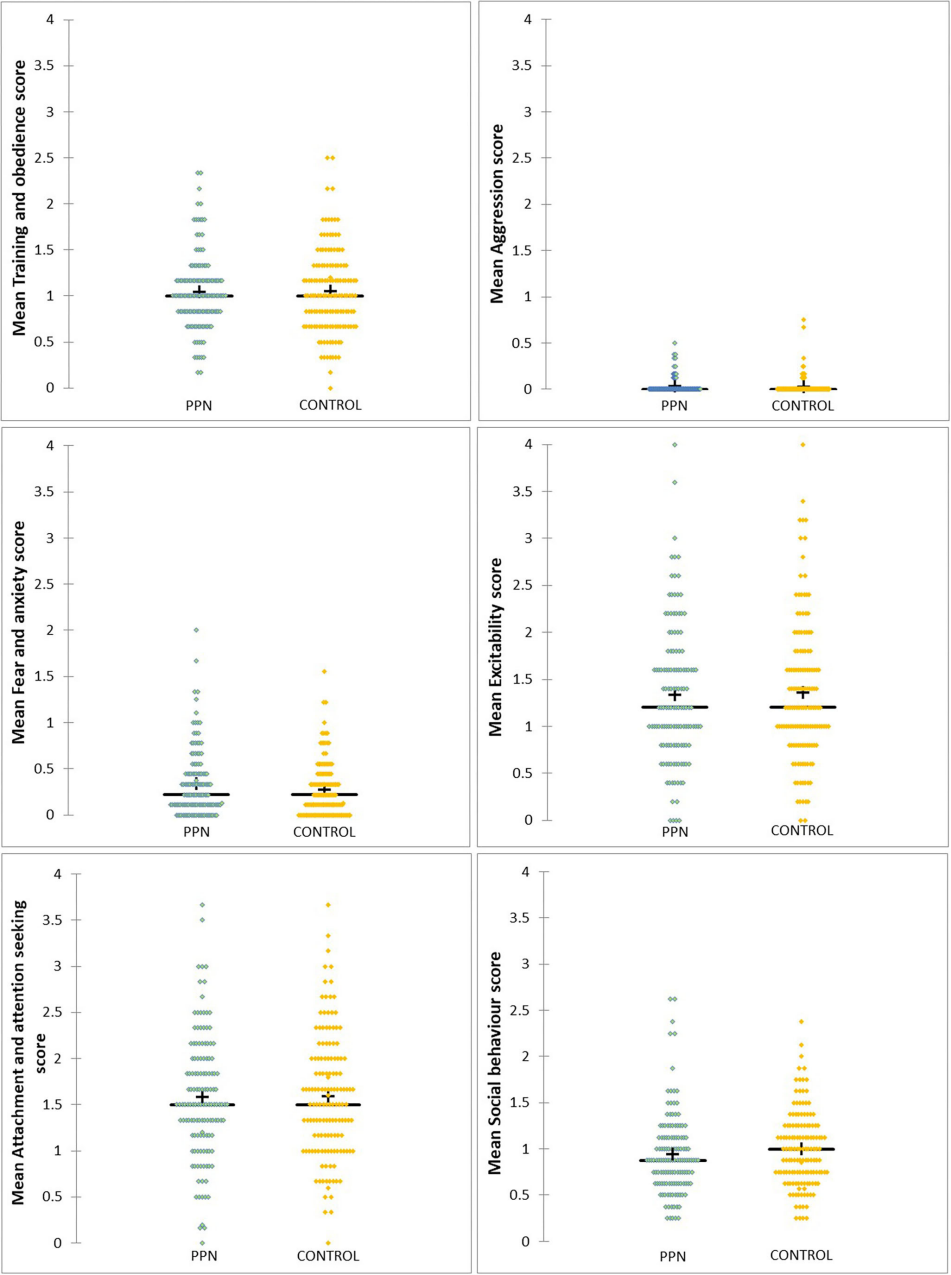
The distribution of mean scores for six behavioural factors measured using questions from the C-BARQ at 1 year of age for bitches neutered before (PPN, *n* = 134) or after (Control, *n* = 140) puberty. Scores of “0” represented a behaviour that had not been observed.

None of the six models were significant, and, for generalised linear models, 91% Wald CI for all factors and covariates included zero. Therefore, mean scores for training and obedience (D.F. = 1, F = 0.661, *p* = 0.417), fear and anxiety (Wald chi-square = 0.197, D.F. = 1, *p* = 0.657), excitability (Wald chi-square = 0.513, D.F. = 1, *p* = 0.474), attachment and attention-seeking (D.F. = 1, F = 0.052, *p* = 0.819), and social behaviour (Wald chi-square = 1.903, D.F. = 1, *p* = 0.168) were not different between PPN and Control bitches and were not influenced by any of the fixed factors or covariates included in the models (Table 2A; Figure 1). Mean aggression factor scores were heavily skewed towards “0” and were “0” for 114 PPN and 122 Control bitches. For the 38 bitches with mean aggression scores higher than “0,” mean scores ranged from 0.13 to 0.75 (Table 2B). There was no effect of group (Wald chi-square = 0.341, D.F. = 1, *p* = 0.559), or any other fixed factors or covariates on whether bitches scored “0” or greater than “0” for aggression.

**Table 2A:** General linear models for training and obedience and attachment and attention-seeking.

**Behavioural factor score**	**PPN mean ± SEM** **(range min-max)**	**Control mean ± SEM** **(range min-max)**	**Effect** **size**	**Degrees of** **freedom**	**F**	**Main effectP**
Training and obedience	1.05 ± 0.04 (0.17–2.33)	1.05 ± 0.04 (0.00–2.50)	−0.004	1	0.661	0.417
Attachment and attention- seeking	1.58 ± 0.06 (0.00–3.67)	1.59 ± 0.06 (0.00–3.67)	0.012	1	0.052	0.819

**Table 2B:** Generalised linear models for gamma distribution for fear and anxiety, excitability and social behaviour and with a logit link function for binary aggression data.

**Behavioural factor score**	**PPN mean ± SEM** **(range min-max)**	**Control mean ± SEM** **(range min-max)**	**Effect size**	**Main effect**	**Model parameter estimates for Control compared to PPN**			
				**P**	**B**	**S.E**.	**Wald Chi-square**	**91% Wald CI**
Aggression	0.03 ± 0.01 (0.00–0.50)	0.03 ± 0.01 (0.00–0.75)	−0.03	0.559	−0.752	1.289	0.341	−2.937, 1.433
Fear and anxiety^*^	0.34 ± 0.03 (0.00–2.00)	0.28 ± 0.03 (0.00–1.56)	−0.22	0.657	−0.145	0.328	0.197	−0.01, 0.411
Excitability	1.33 ± 0.06 (0.00–4.00)	1.36 ± 0.06 (0.00–4.00)	0.03	0.474	−0.182	0.254	0.513	−0.613, 0.249
Social behaviour	0.94 ± 0.04 (0.25–2.63)	1.00 ± 0.03 (0.25–2.38)	0.14	0.168	0.262	0.190	1.903	−0.060, 0.584

* *The model fit was poor with the puppy-raising region included; therefore, results shown are for the model excluding this fixed factor. The results from the general (Table 2A) and generalised (in this Table) linear models used to examine differences between bitches neutered before (PPN, n = 134) or after (Control, n = 140) puberty in six behavioural factors scores using questions from the C-BARQ completed at 1 year of age. P-values and model parameter results shown are for the main effect of neutering before or after puberty, with PPN as the reference group*.

#### Questionnaires at 3 Years of Age

The PPN bitches were neutered between 872 and 972 days before questionnaire completion. The Control bitches were neutered between 617 and 822 days before questionnaire completion. Spearman's rank correlation coefficients suggested weak positive correlations between days from neutering surgery and aggression (*r*_*s*_ = 0.149, *p* = 0.040), fear and anxiety (*r*_*s*_ = 0.191, *p* = 0.008) and attachment and attention-seeking (*r*_*s*_ = 0.156, *p* = 0.032). No relationships were identified between days from neutering and the other three behavioural factors (*r*_*s*_ < 0.110). The PPN and Control bitch scores were clustered in two distinct groups by days from neutering surgery (see Supplementary File 3).

##### Factors Influencing Behavioural Factor Scores at 3-Year Questionnaires

**Figure 2:**
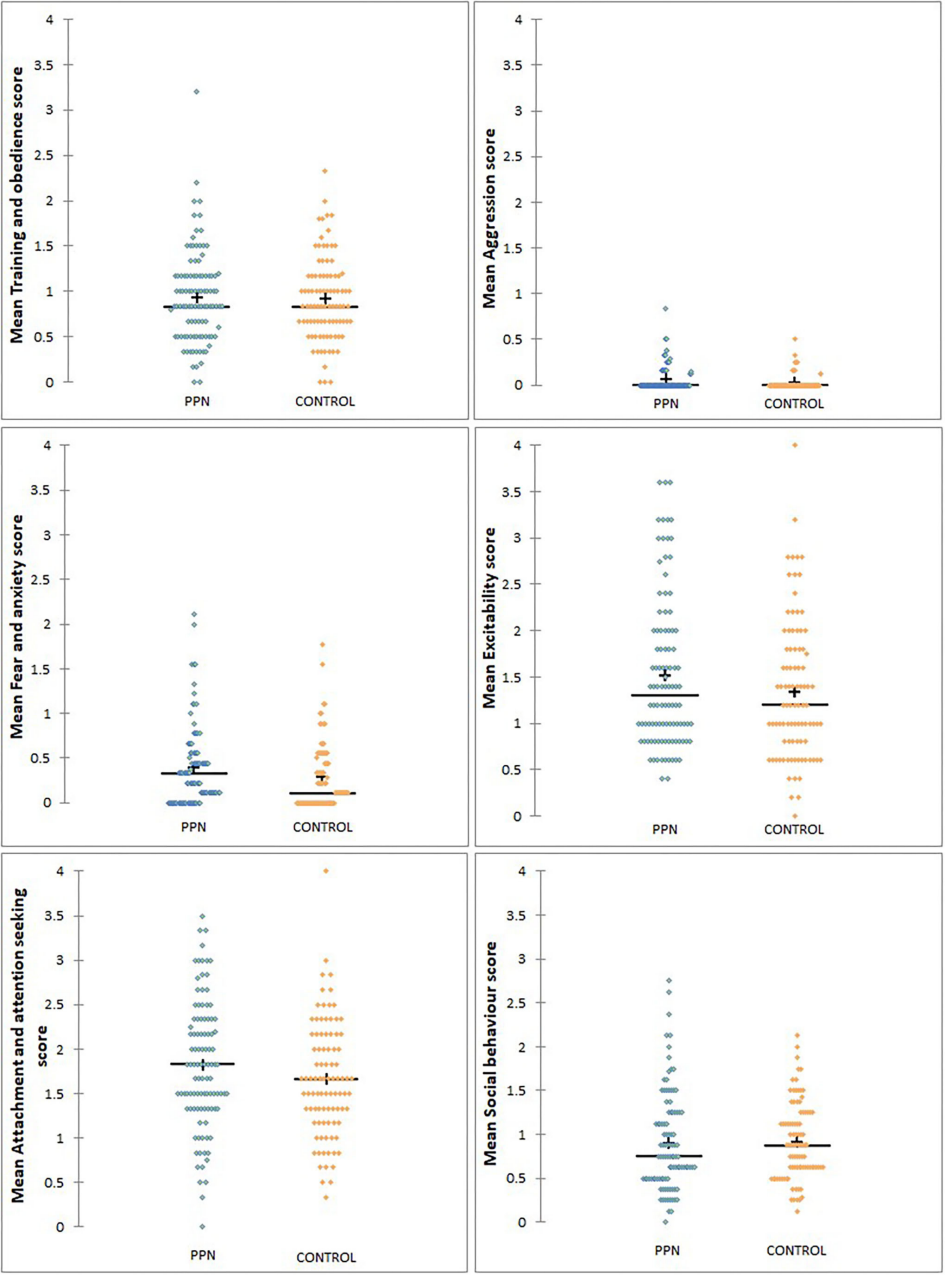
The distribution of mean scores for six behavioural factors measured using questions from the C-BARQ at 3 years of age for bitches neutered before (PPN, *n* = 102) or after (Control, *n* = 91) puberty. Scores of “0” represented a behaviour that had not been observed.

Mean behavioural factor scores were not different between the PPN and Control bitches in any model, and 90% Wald CI for the trial group for all factors included zero (Tables 3A, 3B; Figure 2). The person completing the questionnaire affected mean scores for all the factors, except aggression (Wald chi-square = 3.562, D.F. = 2, *p* = 0.168) and attachment and attention-seeking (D.F. = 2, F = 0.051, *p* = 0.950). *Post-hoc* Tukey's HSD showed that guide dog owners scored significantly lower than rehomers for training and obedience (D.F. = 2, F = 10.985, *p* < 0.001). The guide dog owners were more likely than the rehomers to score bitches lower for fear and anxiety (OR = 1.579, 90% CI = 1.239 to 2.012, *p* = 0.002), excitability (OR = 1.345, 90% CI = 1.167 to 1.551, *p* < 0.001), and social behaviour (OR = 1.610, 90% CI = 1.402 to 1.850, *p* < 0.001). The guide dog owners were also more likely to score bitches lower than puppy raiser rehomers for fear and anxiety (OR = 1.687, 90% CI = 1.231 to 2.311, *p* = 0.006) and social behaviour (OR = 1.290, 90% CI = 1.042 to 1.597, *p* = 0.050). No other fixed effects or covariates influenced any mean behavioural factor scores.

**Table 3A:** General linear models for training and obedience and attachment and attention-seeking.

**Behavioural factor score**	**PPN mean ± SEM** **(range min-max)**	**Control mean ± SEM (range min-max)**	**Effect size**	**Degrees of freedom**	**F**	**Main effectP**
Training and obedience	0.94 ± 0.05 (0.00–3.20)	0.92 ± 0.05 (0.00–2.33)	−0.05	1	1.022	0.313
Attachment and attention- seeking	1.82 ± 0.07 (0.00–3.50)	1.66 ± 0.07 (0.33–4.00)	−0.25	1	0.164	0.686

**Table 3B:** Generalised linear models for gamma distribution for fear and anxiety, excitability, and social behaviour and with a logit link function for binary aggression data.

**Behavioural factor score**	**PPN mean ± SEM** **(range min-max)**	**Control mean ± SEM** **(range min-max)**	**Effect** **size**	**Main** **effect**	**Model parameter estimates for control compared to PPN**			
				**P**	**B**	**S.E**.	**Wald Chi-square**	**90% Wald CI**
Aggression	0.07 ± 0.01 (0.00–0.83)	0.03 ± 0.01 (0.00–0.50)	−0.48	0.995	−0.011	1.815	0.000	−2.997, 2.975
Fear and anxiety	0.40 ± 0.04 (0.00–2.11)	0.29 ± 0.04 (0.00–1.78)	−0.30	0.298	0.567	0.545	1.081	−0.330, 1.464
Excitability	1.52 ± 0.08 (0.40–3.60)	1.35 ± 0.08 (0.00–4.00)	−0.23	0.383	−0.285	0.326	0.761	−0.821, 0.251
Social behaviour	0.91 ± 0.06 (0.00–2.75)	0.92 ± 0.05 (0.13–2.13)	−0.03	0.420	0.253	0.314	0.649	−0.263, 0.770

*The results from the general (Table 3A) and generalised (in this Table) linear models used to examine differences between bitches neutered before (PPN, n = 102) or after (Control, n = 91) puberty in six behavioural factors scores using questions from the C-BARQ completed at 3 years of age. P values and model parameter results shown are for the main effect of neutering before or after puberty, with PPN as the reference group*.

Mean aggression factor scores were heavily skewed towards “0” and were “0” for 159 of the 193 bitches with completed questionnaires (78 PPN, 81 Control). For the 34 bitches with mean aggression scores higher than “0,” the scores were low and ranged from 0.13 to 0.83 (Table 3B). There was no effect of trial group (Wald chi-square =0.000, D.F. = 1, *p* = 0.995), or any other fixed factors or covariates on whether the bitches scored “0” or greater than “0” for aggression.

#### Change in Behavioural Factor Scores Between 1 and 3 Years of Age

**Figure 3:**
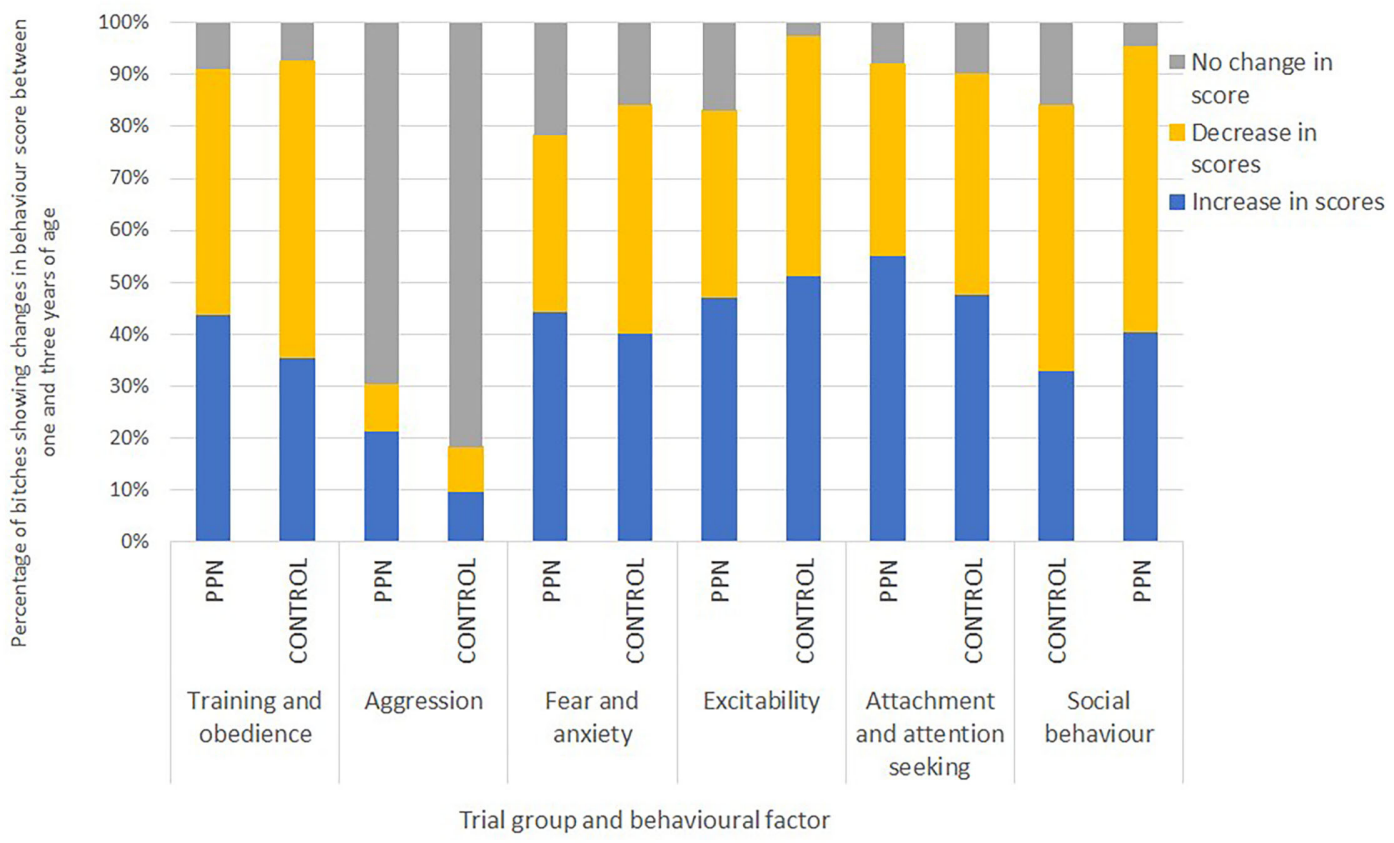
The percentage of bitches neutered before (PPN, *n* = 89) or after (Control, *n* = 82) puberty that had mean factor scores in 3-year behaviour questionnaires that represented more-desirable, the same, or less-desirable behaviour than their scores in 1-year questionnaires for the six behavioural factors.

There was no consistent pattern of increase or decrease in mean scores observed for any trial group or behaviour factor. For each behaviour factor, there were some bitches with mean scores that increased and some with scores that decreased by 3 years of age. An increase in a score (change to less desirable behaviour) appeared more frequently for the PPN than the Control bitches for all the factors, except excitability. A lower proportion of the PPN than the Control bitches had scores that decreased (changed to more desirable behaviour) between 1 and 3 years of age for training and obedience, fear and anxiety, and excitability (Figure 3).

##### Factors Influencing the Change in Behavioural Factor Scores Between 1 and 3 Year Questionnaires

**Figure 4:**
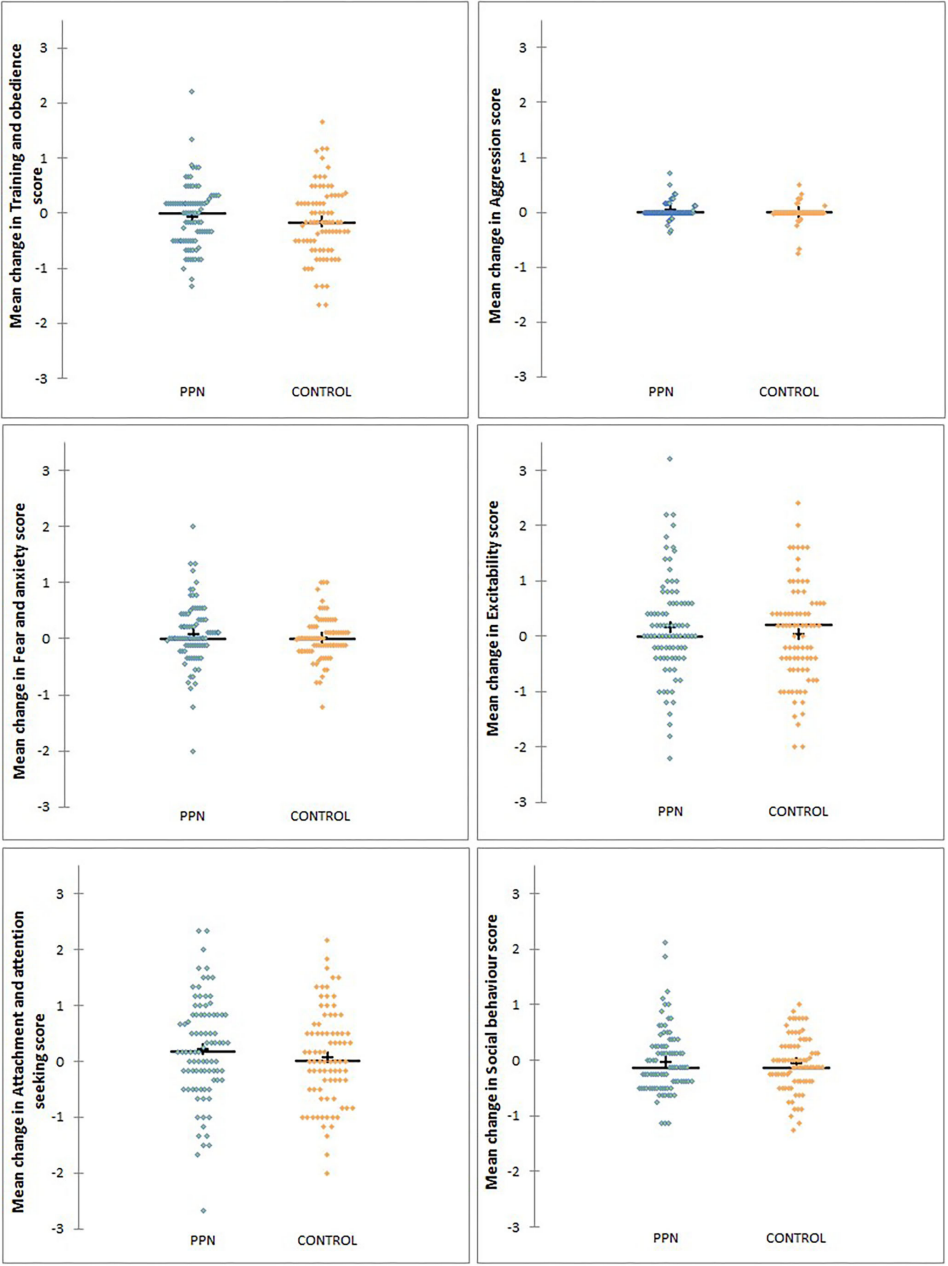
The distribution of changes in mean scores between 1 and 3 years of age for six behavioural factors measured using questions from the C-BARQ for bitches neutered before (PPN, *n* = 89) or after (Control, *n* = 82) puberty.

The change in behaviour factor scores from 1 to 3 years of age was not affected by the trial group for any factor, except aggression (Tables 4A, 4B; Figure 4). There was no significant effect of any of the factors included in the models for training and obedience (D.F. = 5, F = 0.836, *p* = 0.526), excitability (D.F. = 5, F = 1.255, *p* = 0.286), attachment and attention-seeking (D.F. = 5, F = 0.249, *p* = 0.940), and social behaviour (D.F. = 5, F = 1.644, *p* = 0.151).

**Table 4A:** General linear models for training and obedience, excitability, attachment and attention-seeking, and social behaviour.

**Behavioural factor score**	**PPN mean ± SEM** **(range min-max)**	**Control mean ± SEM** **(range min-max)**	**Effect** **size**	**Degrees of freedom**	**F**	**Main effectP**
Training and obedience	−0.07 ± 0.06 (−1.33–2.20)	−0.16 ± 0.07 (−1.67–1.67)	−0.14	1	1.288	0.258
Excitability	0.158 ± 0.10 (−2.20-3.20)	0.03 ± 0.10 (−2.00–2.40)	−0.14	1	0.489	0.485
Attachment and attention-seeking	0.22 ± 0.10 (−2.67–2.33)	0.08 ± 0.10 (−2.00–2.17)	−0.16	1	1.043	0.309
Social behaviour	−0.02 ± 0.06 (−1.13–2.13)	−0.06 ± 0.06 (−1.25–1.00)	−0.08	1	0.204	0.652

**Table 4B:** Generalised linear models for fear and anxiety and aggression.

**Behavioural factor score**	**PPN mean ± SEM** **(range min-max)**	**Control mean ± SEM** **(range min-max)**	**Effect size**	**Main effect**	**Model parameter estimates for control compared to PPN**			
				**P**	**B**	**S.E**.	**Wald Chi-square**	**90% Wald CI**
Aggression	0.04 ± 0.02 (−0.38–0.71)	0.00 ± 0.02 (−0.75–0.50)	−0.26	0.060	−0.042	0.022	3.546	−0.078, 0.005
Fear and anxiety	0.09 ± 0.06 (−2.00–2.00)	0.02 ± 0.04 (−1.22–1.00)	−0.19	0.252	0.083	0.072	1.313	−0.035, 0.201

*The results from the general (Table 4A) and generalised (in this Table) linear models used to examine differences between bitches neutered before (PPN, n = 89) or after (Control, n = 82) puberty, showing changes in six mean behavioural factor scores using questions from the C-BARQ completed at 1 and 3 years of age. P-values and model parameter results shown are for the main effect of neutering before or after puberty, with PPN as the reference group*.

Aggression scores for the Control bitches were less likely to increase (become less desirable) by the 3-year questionnaires than for the PPN bitches (OR = 0.959, 90% CI = 0.924 to 0.995, *p* = 0.06). Mean scores for aggression were “0” at both questionnaires for 127 of the 171 bitches (74.3%; PPN = 61, Control = 66). For 20 PPN and nine Control bitches, the mean scores for aggression increased between 1- and 3-year questionnaires, while the aggression score decreased for eight PPN and eight Control bitches. Effect size was −0.26.

Change in aggression scores was also influenced by breed. The aggression scores for Golden Retriever cross Labradors (mean change =0.03 ±0.14) were more likely to increase (become less desirable) by the 3-year questionnaires than scores for Labrador cross Golden Retrievers (mean change = −0.02 ± 0.20; OR = 1.073, 90% CI = 1.013 to 1.137, *p* = 0.044).

Change in the Fear score was affected by the person completing the 3-year behaviour questionnaire; the scores reported by the rehomers were more likely to increase (become less desirable) than the scores reported by the guide dog owners (OR = 1.319, 90% CI = 1.154 to 1.506, *p* < 0.001).

The majority of the PPN (74.2%) and Control (73.2%) bitches had scores that did not demonstrate a consistent pattern of change between time points across all six factors (Table 5), and there was no difference in the number of bitches with overall increases, decreases or mixed changes in factor scores between the groups (chi-square = 2.727, D.F. = 2, *p* = 0.256).

**Table 5:** The overall direction of change in behaviour scores for six behavioural factors between 1 and 3 years of age measured using questions from the C-BARQ for bitches neutered before (PPN, *n* = 89) or after (Control, *n* = 82) puberty.

**Overall direction of** **change in behavioural** **factor score**	**PPN N**	**PPN %**	**Control N**	**Control %**
Decreased	9	10.1	14	17.1
Increased	14	15.7	8	9.8
Mixed	66	74.2	60	73.2

### Leg Raising When Urinating

Data were available for all 274 bitches at the 1-year questionnaire stage and 188 bitches at the 3-year questionnaire stage. None of the PPN bitches and only two Control bitches were reported to raise their legs when urinating at the 1-year stage: one “seldom” and one “usually.” Questionnaires for both of these bitches were completed before neutering surgery. Six different bitches (four PPN, two Control) were reported to raise their legs when urinating at the 3-year stage, five “sometimes” and one PPN “usually.” Further statistical analysis was not possible due to the low frequency with which this behaviour was reported.

## Discussion

The impacts of neutering before or after puberty on bitch behaviour have not been well-studied. The current study achieved the study aims by investigating the effects of neutering bitches before or after puberty on behaviour in a prospective cohort study design. The results suggest that, for Labrador and Golden Retriever crossbreeds, there are no significant differences in behavioural factor scores measured using questions from the C-BARQ at 1 and 3 years of age in bitches neutered before or after puberty. Additionally, no significant differences were identified to changes in scores from 1 to 3 years of age, with the exception of change in aggression scores. For aggression scores, a significant difference was identified but requires interpretation in terms of effect size and power of the statistical model. These results suggest that prepubertal neutering has no impact (either detrimental or favourable) on future behaviour in female dogs of these breeds. This provides important information for assistance dog organisations and veterinarians to inform neutering policies.

This study used questions from the C-BARQ in a shorter questionnaire, which limits direct comparison between the current study and those using the full version of the C-BARQ due to differences in a subscale structure and may limit the conclusions that can be drawn. This study only included female Labrador and Golden Retriever crossbreeds born and raised in a guide dog programme, which may limit extrapolation of findings to other breeds or populations. At the time of 1-year behaviour questionnaire completion, 91 Control bitches had not yet been neutered, and a further 23 Control bitches were neutered in the two weeks preceding questionnaire completion. The questionnaire respondents were asked to describe how their puppies had been behaving during the last few months; therefore, almost all the respondents were providing responses for entire bitches at 1 year of age. This could be considered as a study limitation; the bitches that had been neutered in the few weeks prior to questionnaire completion may have displayed pain-related behaviours post-surgery, which could have impacted responses to behaviour questions, with the respondents recalling more recent behaviours more readily than behaviour during the previous few months. Additionally, the behaviours displayed by the bitches that were entire at the time of questionnaire completion may have been impacted by an impending oestrus. Whilst there were no differences in behaviour factor scores between the Control bitches that were entire or neutered at the time of behavioural questionnaire completion, future studies that use a similar design could include additional questionnaires at 6 months of age.

The appropriateness of a pet dog questionnaire for guide dog populations has been questioned (55). A new behaviour questionnaire has been developed, which has been proved to be more appropriate for guide dogs than C-BARQ (55, 56). That questionnaire measures behaviours suited to a guide dog programme and demonstrated better predictive power than C-BARQ for questionnaires at 8 months of age (55). Future studies of guide dog programmes could consider using the new guide dog-specific questionnaire; however, this has not yet been validated with adult dogs. In the present study, no questions were asked relating to the bitch's health or events experienced – factors that could influence their behaviour (57–61). Aiming to understand and either control for or exclude dogs based on health conditions or events that may be impacting dog behaviour is advised for future studies.

A selection of existing papers that report C-BARQ results for dogs was reviewed to determine the most appropriate methods of data analysis for comparison between the two study groups. Commonly, actual trait scores and the differences that the authors of these papers would consider to be significant were not reported. No studies have reported the difference in C-BARQ trait scores that would represent a real-life, observable, significant change in dog behaviour, although Hsu and Serpell (52) reported graphical representations for distributions of scores for dogs with and without a clinical behavioural problem. In addition, mean behavioural trait scores and S.D. or actual effect size was rarely reported, which reduced understanding of the size of the difference in scores that are reported as significant.

Power analysis suggested that the detectable effect sizes based on the study population and values of alpha and beta required to balance the likelihood of Types I and II errors were 0.4 to 0.5. Reported or calculated effect sizes from other C-BARQ studies were between 0.1 and 1.2 for training (mean = 0.5), 0.0 and 1.8 for aggression (mean = 0.9), 0.0 and 1.7 for fear (mean = 0.6), 0.3 and 0.7 for excitability (mean, 0.5), and 0.3 and 0.6 for attachment/attention-seeking (mean, 0.4), demonstrating a wide range across different studies (22, 44, 62–64). However, it is more common that effect sizes are not reported and are not possible to determine due to the lack of mean and S.D. data reported.

Based on mean effect sizes that have been reported, and with no prior C-BARQ data for the current study population on which to base effect size estimates, the effect sizes from power analysis appeared suitable. However, actual effect sizes were small, between 0.0 and 0.3 for behaviour questionnaires, with the exception of aggression, which was 0.48 at 3 years of age. The Guide Dogs breeding programme was established in 1960 (65), and dogs have been selectively bred to be behaviourally well-suited for the guide dog programme since then. In addition, the bitches were all reared and socialised following standardised guidelines and will have similar life experiences, which will impact the range of behaviours that may be seen. Indeed, previous work has highlighted that extreme aggressive behaviour is uncommon in guide dogs, and the range of behavioural displays of aggression reported in other dog populations measured using C-BARQ is not observed for the current population (55, 66). This reduced variation could influence the small effect sizes observed. However, direct comparison between the current study, which used some C-BARQ questions, and previous studies, which report results of the original C-BARQ, is limited by the differences in questionnaire structure. Despite this, the findings are useful to inform sample size decisions for future studies. Based on the effect sizes reported in this study, power analysis suggests that around 2,000 dogs would be needed for each trial group in some cases, and, for some behavioural traits, up to 20,000. However, the current study utilised dogs from one of the world's largest assistance dog training programmes, and recruitment of such numbers would not have been possible. Recruitment of dog data from existing data sources, such as from C-BARQ (as in 23) or from large veterinary databases, would allow recruitment of the sample sizes required. However, issues with study design, such as retrospective examination of data, recall bias, limited information about potential confounding and environmental factors, and inconsistent dog rearing and husbandry, would then impact the quality of the results.

The sole previous study that also examined the effects on behaviour of neutering bitches of similar breeds before or after puberty (20) has reported similar findings. In that study, 58 Labrador Retriever bitches of approximately 6 years of age were classified as intact (*n* = 12), neutered before (*n* = 17, median age, 6 months) or after (*n* = 29, median age, 16 months) puberty. No differences in urination behaviours were observed, and no differences in owner-reported urination behaviours or behaviour measured using C-BARQ were identified, although the small number of bitches and the potential for error in classification of timing of neutering due to owner recollection may be considered limitations to the study. The results reported by Balogh et al. (20) and by the present study conflict with the assertions of some authors (e.g., 35–38, 40, 41), but do support the contention that neutering before or after puberty has little impact on behavioural development.

Early neutering has been suggested to increase aggression (7, 17). The significant difference identified between PPN and Control bitches for change in the aggression scores in the present study suggests that the bitches neutered prepubertally were more likely to have scores for aggression that became less desirable by 3 years of age. However, the proportion of bitches for which the scores changed between the two time points was small, with the majority scoring “0” at both time points. This is unsurprising; C-BARQ scores for aggression that are skewed towards zero have previously been reported in the general dog population (45) and in guide dog populations (55). Additionally, at 3-year questionnaires, the mean scores for aggression for both groups were <0.1, and no bitch scored >0.8, suggesting that only very mild aggressive behaviours were observed. Effect size for change in the aggression score was smaller than the suggested effect size based on power analysis. No difference was detected between the PPN and Control bitches in the aggression scores at 3 years of age, and, for that analysis, effect size was larger than the effect size suggested by power analysis. Further investigation of the potential impact of neutering on aggression with larger numbers of dogs could provide more information. However, aggression in guide dogs is known to be infrequently observed (55, 66) and is likely to have been selected against (66, 67). This suggests that, at least for female dogs of these crossbreeds, there is no considerable detrimental impact of neutering before compared to after puberty on observed aggressive behaviour, consistent with findings of other authors (20, 23).

The fear and anxiety scores reported by the rehomers were more likely to increase between 1 and 3 years of age than those reported by the guide dog owners. Fear is one of the major reasons reported for dogs being unsuccessful in guide dog programmes (4, 67, 68). Fear and anxiety was also one of the behavioural factors with scores representing more desirable behaviour more likely to be reported by guide dog owners than rehomers for dogs at 3 years of age, alongside training and obedience, excitability, and social behaviour. Variation in scores between different participant groups could be expected (48, 49). However, within the current study, it was not possible to determine whether the scores that changed to reflect more desirable behaviour reported by the guide dog owners were due to factors relating to the guide dog owners themselves, or due to actual differences in bitch behaviour. Questionnaires completed by the guide dog owners for the bitches that were 3 years of age were reporting on behaviours observed in their working guide dogs. Thus, it could be reasonable to expect more desirable behaviour in this group. Less-desirable C-BARQ scores for aggression, fear, attachment and attention-seeking, excitability, and energy have been reported for dogs that were unsuccessful in a guide dog programme (69).

Behaviour questionnaires that require respondents to recall dog behaviour over a period of time may be subject to recall bias, and any particularly uncharacteristic or undesirable behaviours may be more readily remembered (70). Bias may also result from the respondents' subjective interpretation of behaviour, experience, and knowledge of dog behaviour and body language, and duration of knowing the dog (48, 71, 72). Behaviour questionnaires completed at 1 and 3 years of age were completed by different respondents; the dogs' puppy raisers and guide dog owners or rehomers, which could affect comparisons between time points. Additionally, guide dog owners may not notice behavioural signs or body language, which indicate very mild behaviours, which could have resulted in lower behaviour factor scores. The nature of this study's design means that these risks could be expected to apply to both study groups equally. Conducting a practical behaviour assessment to examine differences in behaviour that would be unaffected by respondent bias could be considered for future studies. However, this was not feasible within the current study due to the wide geographic spread of bitches either working with guide dog owners or rehomed in locations throughout the UK.

Accounting for the reason for neutering when investigating the impacts of neutering on behaviour is important. Podberscek and Serpell (73) reported that the increased aggression identified in neutered dogs compared to entire dogs disappeared when dogs that were neutered because of aggression were removed from the analysis. In the current study, no bitches were neutered for aggression, or other behavioural reasons, and so this contributes data to the small number of studies where neutering for behavioural reasons was considered.

Leg raising whilst urinating was included in this study, following discussion at the 2013 Association of Pet Behaviour Counsellors Annual Conference regarding masculinisation of urination posture in female dogs. This has been proposed to result from the effect of gonadal hormones and has been documented in some bitches exposed to testosterone *in utero* and postnatally (74, 75). In the current study, so few bitches were reported to raise their legs during urination that statistical analysis was not possible. Neutering before puberty did not appear to cause a notable increase in the propensity to adopt a raised leg posture whilst urinating.

In conclusion, this is the first time that the effects on behaviour of neutering bitches before or after puberty have been investigated in a prospective cohort study design. Using questions taken from a validated measure of dog behaviour, the results suggest that neutering bitches in an assistance dog programme before or after puberty has little to no effect on their future behaviour. There are important limitations to consider in regard to the observed effect sizes compared to those that could have been expected when interpreting the results. However, the study provides important information for other researchers in the field in regard to the size of study populations required for valid statistical analysis. Additionally, concerns about possible impacts on behaviour should not inform decisions about the timing of neutering in isolation, but should be considered alongside other evidence relating to the impacts on health and well-being.

These results will be of interest to assistance and working dog organisations, veterinarians, and people working in the field of dog behaviour and training, as well as pet dog owners, as they provide novel information on the impact of neutering relative to the stage of reproductive development on future behaviour of bitches.

## Data Availability Statement

The datasets presented in this article are not readily available because Guide Dogs encourages high quality research to improve the health, temperament and welfare of its dogs. Given the unique nature of this population and the data we collect, Guide Dogs is keen to ensure the integrity of any associated research and prevent misrepresentation. As such, we do not routinely release raw data but will allow its use within high quality research proposals that have been approved under Guide Dogs research governance process. Requests to access the datasets should be directed to canine.research@guidedogs.org.uk.

## Ethics Statement

Ethical review and approval was not required for the study on human participants in accordance with the local legislation and institutional requirements. Written informed consent for participation was not required for this study in accordance with the national legislation and the institutional requirements. The animal study was reviewed and approved by University of Nottingham, School of Veterinary Medicine and Science Committee for Animal Research and Ethics.

## Author Contributions

The idea for the paper was conceived and the final drafts were reviewed by RM, SF, SC, RP, and GE. The experiments were designed by RM, SF, SC, and GE. The experiments were performed and the paper was written by RM. The data were analysed by RM and RP. All authors approved the final version.

## Conflict of Interest

RM was employed by Guide Dogs UK. The remaining authors declare that the research was conducted in the absence of any commercial or financial relationships that could be construed as a potential conflict of interest.

## Publisher's Note

All claims expressed in this article are solely those of the authors and do not necessarily represent those of their affiliated organizations, or those of the publisher, the editors and the reviewers. Any product that may be evaluated in this article, or claim that may be made by its manufacturer, is not guaranteed or endorsed by the publisher.
