# Supplementary material for: A Prospective Cohort Study Investigating the Behavioural Development of Bitches in a Guide Dog Training Programme Neutered Prepubertally or Post-Pubertally
Source: Front Vet Sci. 2022 Jul 7;9:902775. doi: 10.3389/fvets.2022.902775 (PMC9301489; doi:10.3389/fvets.2022.902775)
Supplement: Supplementary file 1 [file Data_Sheet_1.docx]

Supplementary material 1. The questions included in the behaviour questionnaire including the behavioural factor groupings and the answer scales. * denotes questions for which the scores were reversed prior to analysis.

| **Behavioural factor** | **Question** | **Scale** |
| --- | --- | --- |
| **Training and obedience** | When off the leash, returns immediately when called* | **Never, Seldom, Sometimes, Usually, Always** |
|  | Obeys the “sit” command immediately* |  |
|  | Obeys the “stay” command immediately* |  |
|  | Seems to attend/listen closely to everything you say or do* |  |
|  | Slow to learn new tasks |  |
|  | Easily distracted by interesting sights, sounds or smells |  |
| **Aggression** | When approached directly by an unfamiliar person while being walked/exercised on a lead | **5-point scale (0= No aggression, 4= Serious aggression)** |
|  | When toys, bones or other objects are taken away by a household member |  |
|  | During grooming or being given treatments by a household member |  |
|  | When approached directly by a household member while s/he (the dog) is eating or has a toy/favourite object |  |
|  | When approached directly by an unfamiliar dog while being walked/exercised on a lead |  |
|  | Toward unfamiliar persons visiting your home |  |
|  | When approached while eating by another (familiar) household dog (leave blank if no other dogs) |  |
|  | When approached while playing with/chewing a favourite toy, bone, object, etc., by another (familiar) household dog (leave blank if no other dogs) |  |
| **Fear and anxiety** | When approached directly by an unfamiliar person while away from your home | **5-point scale (0=No fear, 4=Extreme fear)** |
|  | In response to sudden or loud noises (e.g. vacuum cleaner, car backfire, road drills, objects being dropped, etc.) |  |
|  | In heavy traffic |  |
|  | When examined/treated by a veterinarian |  |
|  | During thunderstorms, firework displays, or similar events |  |
|  | When approached directly by an unfamiliar dog of the same or larger size |  |
|  | When approached directly by an unfamiliar dog of a smaller size |  |
|  | In response to wind or wind-blown objects |  |
|  | When groomed or bathed by a household member |  |
| **Excitability** | When you or other members of the household come home after a brief absence | **5-point scales (0 = calm, 4 = extremely excitable)** |
|  | When playing with you or other members of your household |  |
|  | Just before being taken for a walk |  |
|  | Just before being taken on a car trip |  |
|  | When visitors arrive at your home |  |
| **Attachment and attention-seeking** | Displays a strong attachment for one particular member of the household | **Never, Seldom, Sometimes, Usually, Always** |
|  | Tends to follow you (or other members of household) about the house, from room to room |  |
|  | Tends to sit close to, or in contact with, you (or others) when you are sitting down |  |
|  | Tends to nudge, nuzzle or paw you (or others) for attention when you are sitting down |  |
|  | Becomes agitated (whines, jumps up, tries to intervene) when you (or others) show affection for another person |  |
|  | Becomes agitated (whines, jumps up, tries to intervene) when you (or others) show affection for another dog or animal |  |
| **Social behaviour** | Chases or would chase birds or small animals, such as cats or rabbits, given the opportunity | **Never, Seldom, Sometimes, Usually, Always** |
|  | Rolls in animal droppings or other ‘smelly’ substances |  |
|  | Eats own or other dog’s faeces |  |
|  | Chews inappropriate objects |  |
|  | ‘Mounts’ objects, furniture, or people |  |
|  | Begs persistently for food when people are eating |  |
|  | Steals food |  |
|  | Playful, puppyish, boisterous |  |
| **Additional question** | Does your dog ever raise their leg during spending [when urinating] | **Never, Seldom, Sometimes, Usually, Always** |
