# Supplementary material for: A Prospective Cohort Study Investigating the Behavioural Development of Bitches in a Guide Dog Training Programme Neutered Prepubertally or Post-Pubertally
Source: Front Vet Sci. 2022 Jul 7;9:902775. doi: 10.3389/fvets.2022.902775 (PMC9301489; doi:10.3389/fvets.2022.902775)

Supplementary material 3. Scatterplots showing relationships between the six behavioural factor scores measured using questions from the C-BARQ at three-years of age and the days between neutering surgery and questionnaire completion for bitches in the prospective cohort study examining the effect of neutering before (PPN, n=102) or after (Control, n=91) puberty.


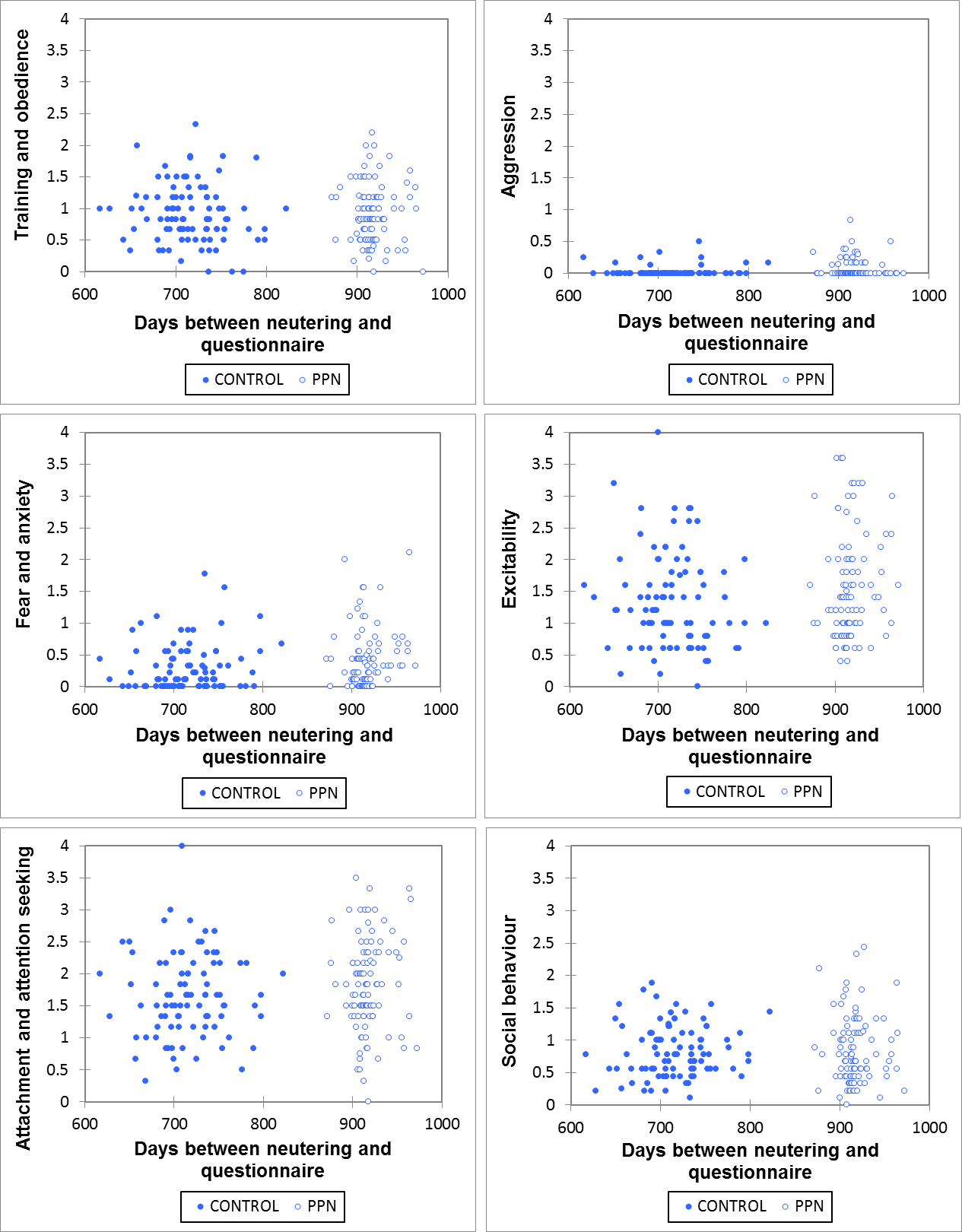

Supplement: Supplementary file 3 [file Data_Sheet_3.docx]
